# Unveiling the role of disulfidptosis-related genes in the pathogenesis of non-alcoholic fatty liver disease

**DOI:** 10.3389/fimmu.2024.1386905

**Published:** 2024-05-15

**Authors:** Xiaohua Luo, Junjie Guo, Hongbo Deng, Zhiyong He, Yifan Wen, Zhongzhou Si, Jiequn Li

**Affiliations:** Department of Liver Transplant, The Second Xiangya Hospital, Central South University, Changsha, China

**Keywords:** NAFLD, disulfidptosis, oxidative stress, Kupffer cell, MYL6

## Abstract

**Backgrounds:**

Non-alcoholic fatty liver disease (NAFLD) presents as a common liver disease characterized by an indistinct pathogenesis. Disulfidptosis is a recently identified mode of cell death. This study aimed to investigate the potential role of disulfidptosis-related genes (DRGs) in the pathogenesis of NAFLD.

**Methods:**

Gene expression profiles were obtained from the bulk RNA dataset GSE126848 and the single-cell RNA dataset GSE136103, both associated with NAFLD. Our study assessed the expression of DRGs in NAFLD and normal tissues. Weighted gene co-expression network analysis (WGCNA) and differential expression analysis were employed to identify the key NAFLD-specific differentially expressed DRGs (DE-DRGs). To explore the biological functions and immune regulatory roles of these key DE-DRGs, we conducted immune infiltration analysis, functional enrichment analysis, consensus clustering analysis, and single-cell differential state analysis. Finally, we validated the expression and biological functions of DRGs in NAFLD patients using histology and RNA-sequencing transcriptomic assays with human liver tissue samples.

**Results:**

Through the intersection of WGCNA, differentially expressed genes, and DRGs, two key DE-DRGs (DSTN and MYL6) were identified. Immune infiltration analysis indicated a higher proportion of macrophages, T cells, and resting dendritic cells in NAFLD compared to control liver samples. Based on the key DE-DRGs, Two disulfidptosis clusters were defined in GSE126848. Cluster 1, with higher expression of the key DE-DRGs, exhibited increased immune infiltration abundance and was closely associated with oxidative stress and immune regulation compared to cluster 2. High-resolution analysis of mononuclear phagocytes highlighted the potential role of MYL6 in intrahepatic M1 phenotype Kupffer cells in NAFLD patients. Our transcriptome data revealed that the expression levels of the majority of DRGs were significantly increased in NAFLD patients. NAFLD patients exhibit elevated MYL6 correlating with inflammation, oxidative stress, and disease severity, offering promising diagnostic specificity.

**Conclusion:**

This comprehensive study provides evidence for the association between NAFLD and disulfidptosis, identifying potential target genes and pathways in NAFLD. The identification of MYL6 as a possible treatment target for NAFLD provided a novel understanding of the disease’s development.

## 1 Introduction

Non-alcoholic fatty liver disease (NAFLD) comprises a collection of chronic liver disorders distinguished by the excessive accumulation of lipids within hepatocytes and the presence of steatosis (1). The global occurrence of this condition has significantly risen over the years, making NAFLD the prevailing liver ailment across the globe (2). It is currently estimated to affect up to 25% of the population (3). It covers a range of liver conditions that vary from simple steatosis (non-alcoholic fatty liver, NAFL) to non-alcoholic steatohepatitis (NASH). Increasing epidemiological evidence suggests that NAFLD is quickly becoming a primary cause of hepatocellular carcinoma (HCC) in numerous instances (4, 5). Despite the increasing prevalence of NAFLD over time, its underlying causes remain unclear and there is a lack of definitive treatment options (6). Therefore, it is critically important to identify potential pathogenic and therapeutic targets in NAFLD.

Recent research has identified a novel metabolism-related regulated cell death mechanism termed disulfidptosis (7). Xiaoguang Liu et al. discovered that when the intracellular reducing molecule nicotinamide adenine dinucleotide phosphate was depleted, leading to the accumulation of disulfide compounds such as cystine and triggering disulfide stress. This led to the creation of disulfide bonds between cytoskeletal proteins and the collapse of the actin filament network, ultimately causing disulfidptosis to occur (7). In this process, the cellular redox state played a crucial role. It was widely recognized that individuals with NAFLD demonstrate hepatic oxidative stress caused by compromised mitochondrial respiratory capacity and proton leakage (8). Oxidative stress in NAFLD can induce the activation of Kupffer cells, leading to their polarization towards the pro-inflammatory M1 phenotype. This polarization causes the discharge of many pro-inflammatory cytokines, promoting an inflammatory reaction that harms liver cells and speeds up the advancement of NAFLD (9, 10). Furthermore, previous studies have indicated a close association between sulfide metabolism and the development of NAFLD (11, 12). All of these findings indicated that disulfidptosis-related genes (DRGs) might carry a crucial component in the development and progression of NAFLD.

In this research, we employed weighted gene co-expression network analysis (WGCNA) and differential expression analysis to identify 312 differentially expressed genes (DEGs) strongly associated with NAFLD, elucidating their enriched biological pathways. We also investigated the immune microenvironment in NAFLD patients. Subsequently, we identified two key NAFLD-specific differentially expressed DRGs (DE-DRGs), MYL6 and DSTN, based on 23 DRGs (7). We characterized two subtypes of disulfidptosis associated with NAFLD and immune infiltration. Gene set enrichment analysis (GSEA) results showed that oxidative stress pathways, triglyceride metabolism pathways, and pro-inflammatory pathways were activated in the subtype with high expression of key DE-DRGs. Furthermore, MYL6 was found to be highly expressed in liver immune cells and might potentially promote Kupffer cell polarization towards the pro-inflammatory M1 phenotype through oxidative stress. To reinforce our findings, we further validated the expression of the 23 DRGs in NAFLD patients and explored the associated biological functions and clinical significance of MYL6.

## 2 Materials and methods

### 2.1 Gene expression profiles collection

**Figure 1:**
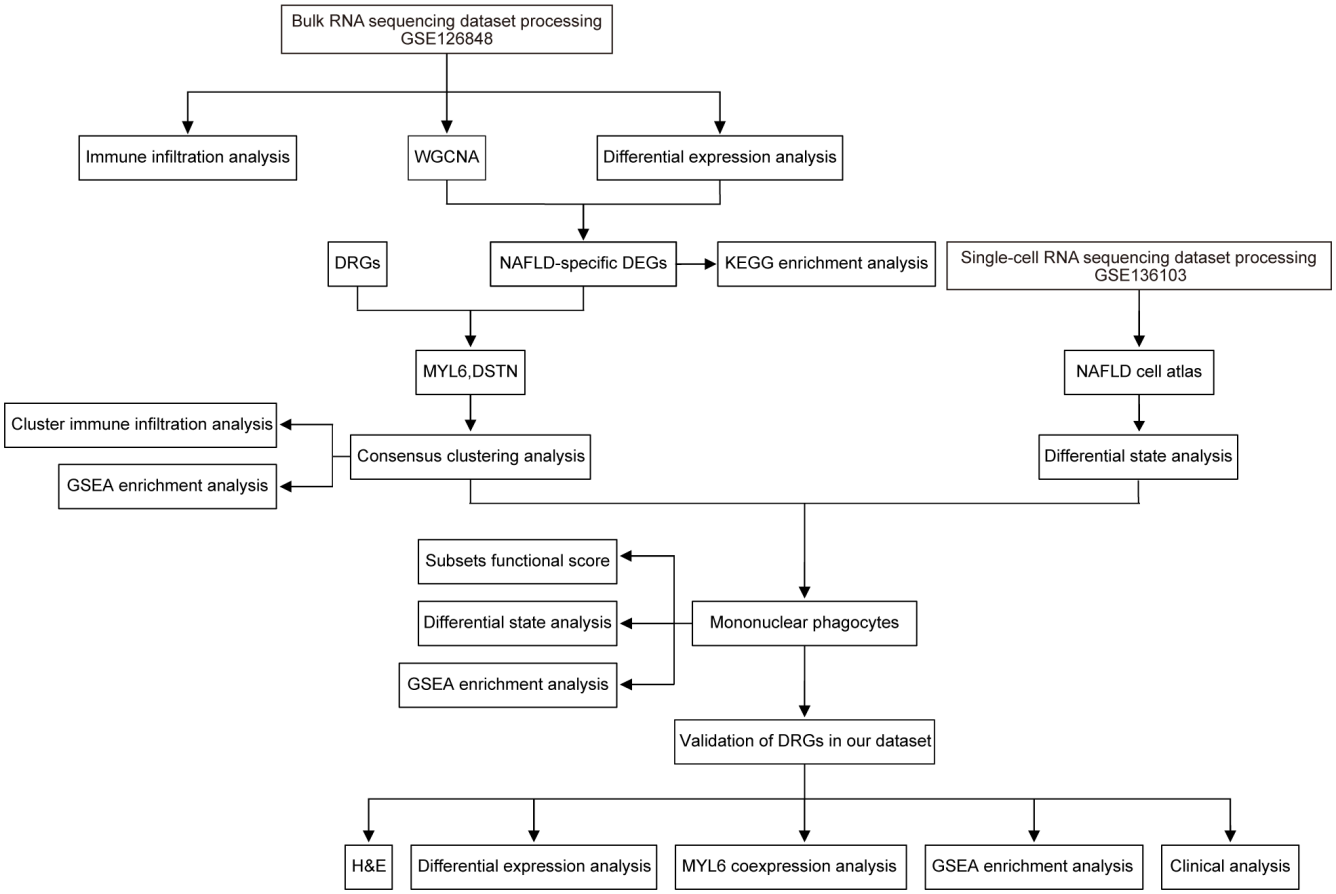
Research design flow chart.

The workflow is shown in **Figure 1**. Original bulk RNA sequencing (RNA-seq) data was collected from the gene expression profile GSE126848, based on the GPL18573 Illumina NextSeq 500 (Homo sapiens) downloaded from the Gene Expression Omnibus (GEO) database, a public depository database of gene expression data (13). Data of single-cell RNA sequencing (scRNA-seq) from GSE136103 were obtained from the GEO database (14). The GSE136103 dataset included single-cell transcriptome data from 11 healthy patient liver tissue samples, 9 cirrhotic patient liver tissue samples, 4 cirrhotic patient PBMC samples, and 2 mouse liver samples. Only 4 human NAFLD-associated cirrhotic samples were included, so all of them were included in our study as the NAFLD group and 4 human healthy liver tissue samples were randomly selected as the control group. Additional information regarding the aforementioned datasets is provided in **Supplementary Table S1**.

### 2.2 Weighted gene co-expression network analysis

We divided genes into different modules based on their similar expression in 57 liver samples using the WGCNA method (15). After evaluating the significance of genes in the module and analyzing the correlation between modules and subtypes, a module strongly associated with NAFLD was discovered. The genes within this module were then utilized for further investigation.

### 2.3 Differential expression analysis

The RNA-seq data was analyzed for differentially expressed genes (DEGs) using the DESeq2 R package (version 1.40.2) (16). DEGs were classified as significant if their absolute log2 fold change (log2FC) was greater than 0.5 and their *p-value* was less than 0.05. Ensemble ID with no corresponding gene symbols were removed, and genes with more than one Ensemble ID were averaged.

### 2.4 Functional enrichment analysis

With the “clusterProfiler” package (version 4.8.1), we conducted GSEA and the Kyoto Encyclopedia of Genes and Genomes (KEGG) pathway enrichment analyses (17, 18). The “c2.cp.v2023.2.Hs.symbols.gmt” was used as the annotation gene set (19). Significant terms were only considered if their *p-value* was less than 0.05. The outcomes of the enrichment analyses were graphically represented through the use of the “clusterProfiler” package (20), “enrichplot” package (version 1.20.0), and “GseaVis” package (version 0.0.9) in R 4.3.1 software.

### 2.5 Immune infiltration analysis

In our research, we utilized three algorithms, CIBERSORT, xCell, and ssGSEA, to quantify immune cell infiltration in NAFLD liver tissues (21–23). We assessed the relative proportions of immune cells in each sample. Additionally, we employed Spearman correlation analysis to assess the correlation between the relative proportions of different cells in CIBERSORT and the mRNA levels of key DE-DRGs. Finally, we visualized the results of immune infiltration analysis using the R package “ggplot2” (version 3.4.4).

### 2.6 Consensus clustering analysis for the key DE-DRGs

Liu’s study provided the information for 23 DRGs (SLC7A11, CD2AP, PDLIM1, ACTN4, MYH10, IQGAP1, FLNA, TLN1, MYL6, ACTB, CAPZB, GYS1, NDUFA11, NCKAP1, NDUFS1, RPN1, NUBPL, SLC3A2, INF2, MYH9, FLNB, DSTN, LRPPRC) (7). The “RCircos” package (version 1.2.2) was utilized to map the positioning of DRGs on chromosomes. Subsequently, the key DE-DRGs were identified from the DEGs, the module of WGCNA, and the DRGs using an interactive Venn diagram. Consensus clustering analysis was conducted using the R package “ConsensusClusterPlus” (version 1.64.0) to categorize patients into distinct clusters according to the expression levels of the key DE-DRGs. The range of the cluster category’s k value was between 2 and 9. Differences in expression of key DE-DRGs between clusters as reflected by heat map.

### 2.7 Analysis of scRNA-seq data

To identify reliable cell subpopulations from scRNA-seq data, we employed the “Seurat” package (version 4.3.0.1) for data processing (24). Using the Seurat R package, we applied normalization, clustering, dimensionality reduction, and visualization techniques to analyze the scRNA-seq data. Each gene must be expressed in a minimum of three cells. The PercentageFeatureSet function was used to calculate the proportions of mitochondria and ribosomal RNA, making sure that each cell had over 200 genes and less than 6,000 genes with less than 25% mitochondrial content and less than 50% ribosome content per cell. To eliminate the influence of confounding factors, the CellCycleSoring function was utilized. To correct batch effects, we employed the RunHarmony function from the harmony R package using the PCA reduction method to integrate samples (25). We used the FindAllMarkers function to identify DEGs for determining the cellular identity. We utilized a three-step workflow including automatic cell annotation, manual cell annotation and verification. The SingleR package (version 2.2.0) is used for automatic annotation. Marker genes used to define different cell types were selected based on the literature, and these cells were identified by their typical characteristic gene profiles (26–28). Finally, we validated the annotation results with the online tool CellMaker (29). During the examination of each cell type, any group of cells that exhibit the presence of multiple markers from various cell types will be eliminated. The condition-specific responses of cell subpopulations measured from two groups of patients were investigated using the “muscat” package (version 1.15.1) through differential state analysis (30). To demonstrate the functional characteristics of mononuclear phagocytes (MPs), we gathered gene collections from various sources and computed functional scores for each individual cell (28, 31–34). The gene list scores were calculated using the AddModuleScore function in Seurat. Online **Supplementary Table S2** contained detailed information for each score. Finally, certain R packages were used for the visualization of single-cell data results, such as the “plot1cell”, “Nebulosa”, “scRNAtoolVis” and “ggplot2” packages (35–37).

### 2.8 Clinical samples and patient data

This study involved 16 patients who underwent surgical procedures, with six diagnosed with NAFL, four with NASH, and six serving as controls (**Supplementary Table S3**). Two experienced hepatopathologists, who were unaware of clinical data, centrally evaluated liver specimens from all of these patients. Liver samples were assessed for histologic characteristics using the NAFLD activity score (NAS) (38). This research received approval from the Ethics Committee of the Second Xiangya Hospital of Central South University (No. 2019–050). Participant consent was obtained in writing prior to participating in the study.

### 2.9 Hematoxylin and eosin staining of liver tissue

In the initial step, liver tissue samples were fixed using 4% paraformaldehyde. After fixation, the tissues were dehydrated in low to high concentrations of ethanol. Following dehydration, the tissues were transparent in xylene and then embedded in paraffin wax for fixation. Afterward, the tissue was sliced to a thickness of 3–5 um, baked for about 1 h, immersed in xylene to deparaffinize, and then hydrated in ethanol from high to low concentration. Once drained, the slices were placed in a vat of hematoxylin staining solution for a duration of 5 minutes, followed by a thorough rinse with running water to restore their blue color. The sections were then immersed in an eosin vat for 5 min and rinsed again under running water. Following a successful staining process, the sections underwent dehydration using varying concentrations of ethanol, followed by transparency achieved with xylene. Subsequently, the sections were sealed and finally examined and captured using a microscope.

### 2.10 Liver transcriptome sequencing

The RNAmini kit (Qiagen, Germany) was used to extract total RNA from every liver tissue sample. The quality of RNA was assessed using gel electrophoresis and Qubit (Thermo, Waltham, MA, USA). The TruSeq RNA sample preparation kit (Illumina, San Diego, CA, USA) was used to construct strand-specific libraries. The Illumina Novaseq 6000 instrument was used for sequencing, which was performed by Genergy Biotechnology Co. Ltd. (Shanghai, China). Skewer was used to process the raw data and FastQC v0.11.2 was utilized to verify the quality of the data. The length of the read was 2×150 base pairs. *STAR* (version 2.5.3a) was employed to align the clean reads to the Human genome hg38. StringTie software (version 1.3.1c) was utilized to count the raw sequence counts of established genes in all samples.

### 2.11 Statistical analysis

Statistical analysis was performed using R version 4.3.1 and GraphPad Prism 9.3.0 software. The Wilcoxon test was utilized to complete the comparative analysis among the groups. Correlation analysis was conducted using Spearman analysis. The gene expression levels in this study were reported as log2(TPM+1) unless otherwise specified. The statistical difference between the sets was significant with a *p-value* less than 0.05.

## 3 Results

### 3.1 Building co-expression networks and screening of critical module

**Figure 2:**
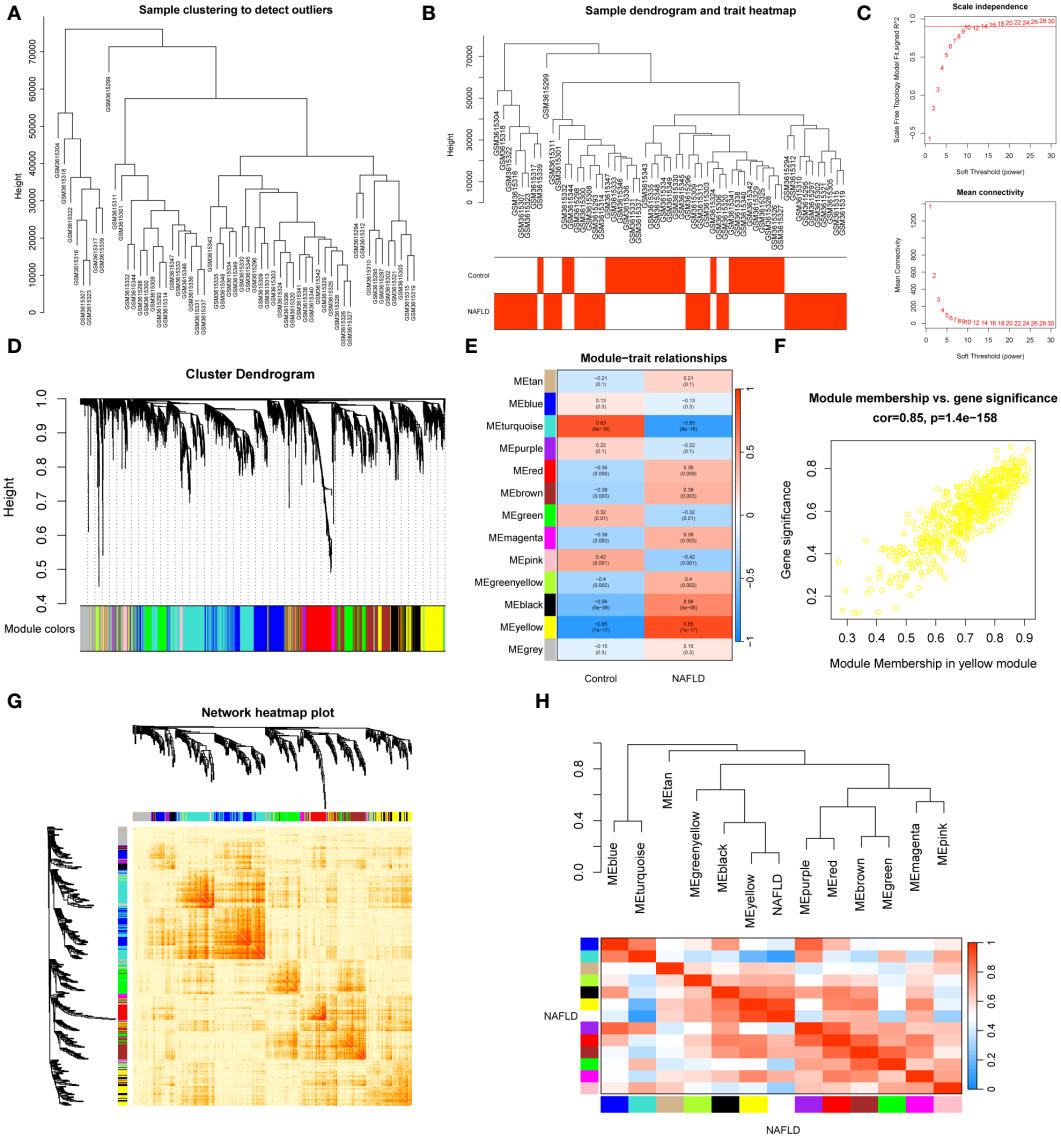
WGCNA analysis and clinically significant module identification. **(A)** Sample clustering dendrogram for outlier detection. **(B)** Clustering dendrogram of 57 liver samples with trait heatmap. **(C)** Under different soft thresholds, the scale-free fit index and mean connectivity are shown. **(D)** Hierarchical clustering of genes in the dendrogram. **(E)** The correlation between clinical traits and gene modules is represented as a heatmap. **(F)** A scatter plot of the yellow module’s eigengenes. **(G)** The co-expression modules are plotted as a network heatmap. **(H)** The eigengene network shows the relationships between modules and clinical trait weights.

In dataset GSE126848, we analyzed 57 liver tissue samples by applying median absolute deviation to screen the top 5000 genes for WGCNA analysis. A clustering tree for the dataset was established and no outliers were found (**Figure 2A**). A dendrogram was formed by clustering samples with clinical traits (**Figure 2B**). Afterwards, we assessed the fitting index for scale-free networks and the average connectivity for different soft threshold powers, using the scale-free R^2^ as a basis (**Figure 2C**). Ultimately, we identified 13 modules through hierarchical clustering (**Figure 2D**). We utilized a correlation heatmap to investigate the correlation between each module and NAFLD. Our analysis revealed that the yellow module exhibited the strongest connection with NAFLD, displaying a correlation value of 0.85 and a *p-value* of 7E-17 (**Figure 2E**). **Figure 2F** showed that the yellow module genes exhibited a strong correlation not only with the yellow module itself but also with NAFLD. A heatmap was constructed to display the network of gene co-expression (**Figure 2G**). **Figure 2H** displayed the connections between the modules and the clinical trait weight. Therefore, we focused on the 564 genes in the yellow module in our follow-up study (**Supplementary Table S4**).

### 3.2 Identification of NAFLD-specific DEGs and their KEGG enrichment analysis

**Figure 3:**
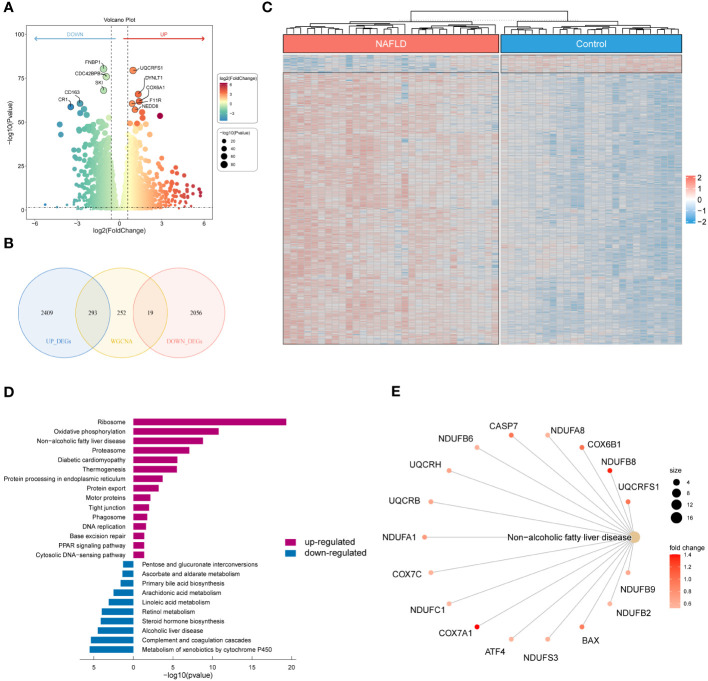
Identification of NAFLD-specific DEGs and their KEGG enrichment analysis. **(A)** Volcano plot of DEGs for the GSE126848 dataset. The top 5 most significantly up- and down-regulated DEGs are labeled individually. **(B)** Venn diagram showing the NAFLD-specific DEGs common to DEGs and WGCNA. **(C)** Heatmap of NAFLD-specific DEGs expression data. **(D)** Bar chart of KEGG enrichment analysis of NAFLD-specific DEGs. **(E)** Network diagram of pathway enrichment analysis.

We examined the DEGs in the GSE126848 dataset to investigate the underlying gene expression pattern and possible correlation between control and NAFLD. By removing the duplicate and irrelevant genes, we discovered a combined count of 2702 up-regulated DEGs and 2075 down-regulated DEGs (**Supplementary Table S5**). **Figure 3A** displayed volcano maps of the DEGs. Subsequently, we identified 312 NAFLD-specific DEGs in DEGs and WGCNA, of which 293 were up-regulated and 19 were down-regulated (**Figure 3B**). We used a heat map to show the expression of NAFLD-specific DEGs in the two groups of samples (**Figure 3C**). In addition, KEGG pathway enrichment analysis was used in R to determine the pathways involved in NAFLD-specific DEGs (**Figure 3D**; **Supplementary Table S6**). The up-regulated NAFLD-specific DEGs were implicated in Ribosome, Oxidative phosphorylation, Non-alcoholic fatty liver disease, Diabetic cardiomyopathy, Motor proteins, Phagosome, and PPAR signaling pathways. Conversely, the down-regulated NAFLD-specific DEGs participated in signaling pathways related to Pentose and glucuronate interconversions, Arachidonic acid metabolism, Linoleic acid metabolism, and Retinol metabolism. Interestingly, we found that 17 of the screened NAFLD-specific DEGs were significantly enriched in the Non-alcoholic fatty liver disease signaling pathway (**Figure 3E**). This confirms the effectiveness of our screening method and also identifies novel genes related to NAFLD. Overall, the NAFLD-specific DEGs identified in this study might be involved in the occurrence and progression of NAFLD.

### 3.3 The immune microenvironment in patients with NAFLD

**Figure 4:**
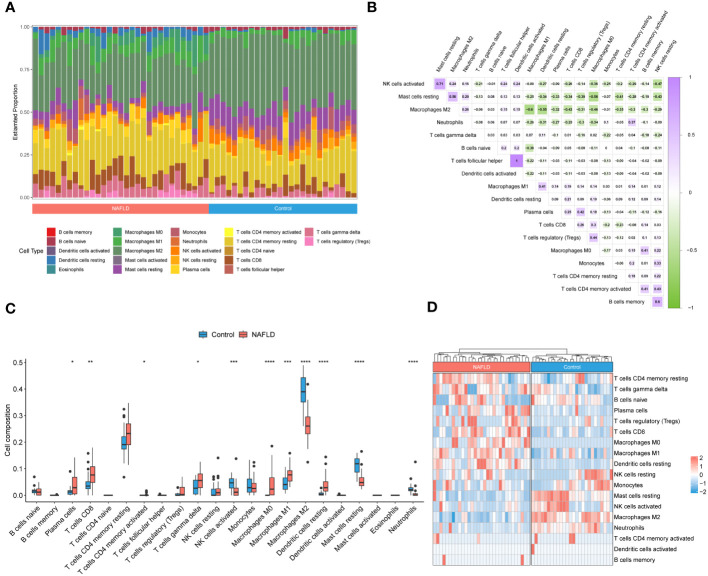
Identification of the immune microenvironment in patients with NAFLD by CIBERSORT algorithm. **(A)** The relative ratio of immunocytes in patients with NAFLD and controls. **(B)** Proportional correlation heatmap of immune cells. The gradient color represents the correlation strength. **(C)** A box plot comparing the infiltration rates of 22 immune cells between NAFLD patients and controls. **(D)** Heatmap of the relative proportion of immune cells in the samples. *P < 0.05, **P < 0.01, ***P < 0.001, ****P < 0.0001.

To investigate the immune microenvironment in patients with NAFLD, we applied to three algorithms, CIBERSORT, xCell, and ssGSEA, to revealed relative immune cell composition (**Figure 4**; **Supplementary Figure S1**). The findings indicated that there is a variation in the immune microenvironment between patients with NAFLD and those without the condition (**Figure 4A**; **Supplementary Table S7**). In addition, there was a close interplay between immune cells, with a significant negative correlation between M1 macrophages and M2 macrophages, and a strong positive correlation between activated dendritic cells and follicular helper T cells (**Figure 4B**). In patients with NAFLD, the proportion of plasma cells, CD8 T cells, CD4 memory activated T cells, gamma delta T cells, M0 macrophages, M1 macrophages, and resting dendritic cells was found to be higher than those in control samples. However, the percentage of activated NK cells, M2 macrophages, resting mast cells, and neutrophils was lower in NAFLD samples (**Figures 4C, D**; **Supplementary Figure S1**). All of these results suggested that alterations in the immune microenvironment play a crucial role in the pathogenesis of NAFLD.

### 3.4 Identification of key DE-DRGs in NAFLD and disulfidptosis clusters immune infiltration analysis

**Figure 5:**
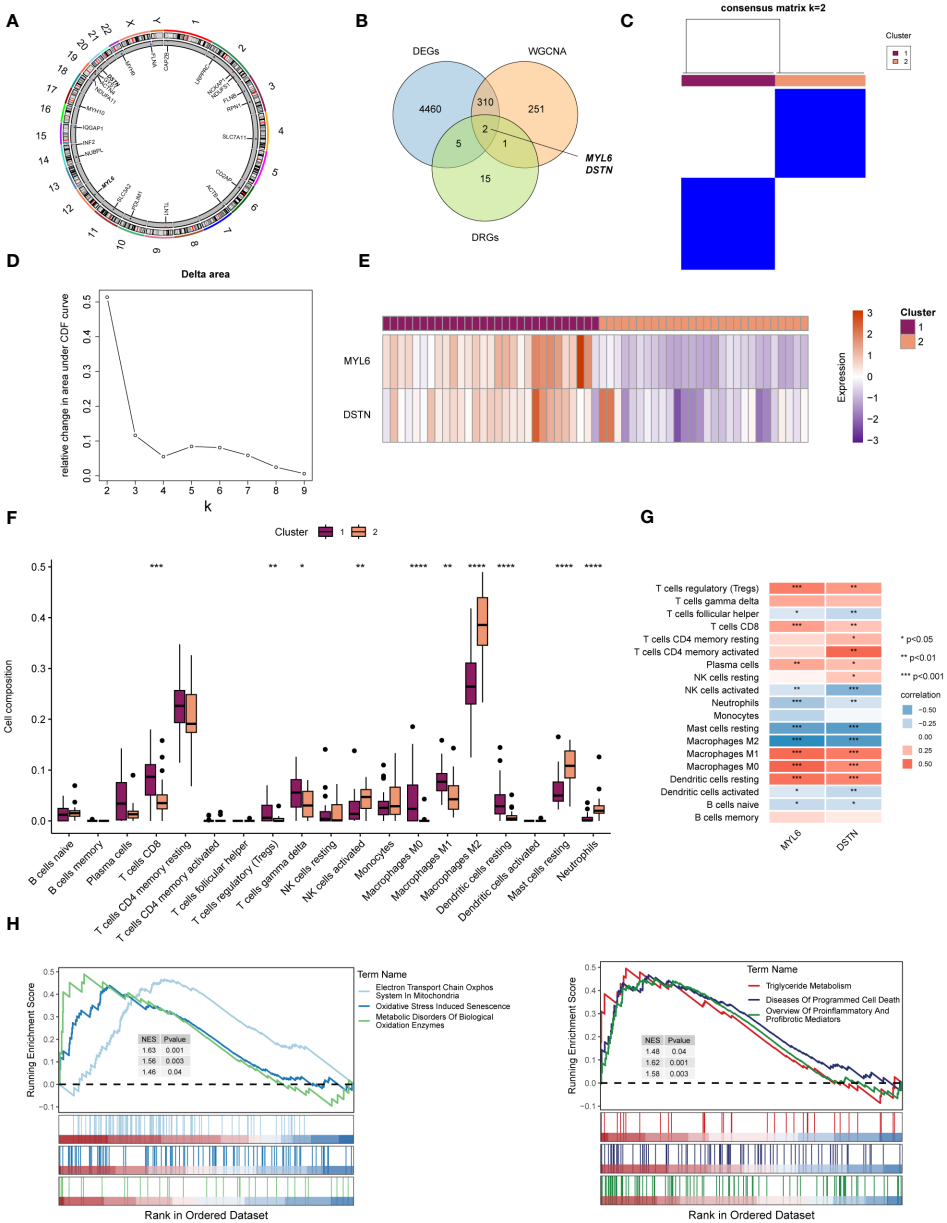
Identification of the key DE-DRGs in NAFLD. **(A)** Chromosome position of the 23 DRGs. **(B)** Venn diagram showing the overlapping genes common to DRGs, DEGs, and WGCNA. **(C)** Consensus matrix plots of the key DE-DRGs. **(D)** Delta area plot. **(E)** Heatmap of relative expression distribution of key DE-DRGs in two clusters. **(F)** Box plot showing immune infiltration differences between disulfidptosis clusters by CIBERSORT algorithm. **(G)** Correlation analysis of the key DE-DRGs with immune cells. **(H)** GSEA analysis based on the canonical pathways gene sets. *P < 0.05, **P < 0.01, ***P < 0.001, ****P < 0.0001.

The location of 23 DRGs on chromosomes were demonstrated in **Figure 5A**. To explore the role of DRGs in NAFLD, we examined the expression patterns of 23 DRGs in the control and NAFLD groups. **Figure 5B** showed that 7 DRGs were differentially expressed in the two groups. After intersecting with the yellow module genes obtained from WGCNA, two key DE-DRGs associated with NAFLD, MYL6 and DSTN, were identified. We then grouped the samples using a consistent clustering algorithm based on the key DE-DRGs. Two disulfidptosis clusters were obtained by cluster analysis (**Figures 5C, D**). Compared to cluster 2, the key DE-DRGs were up-regulated in cluster 1 (**Figure 5E**). To clarify the characteristics of the two disulfidptosis clusters in the immune microenvironment, an immune infiltration analysis was performed (**Figure 5F**). It is worth noting that the analysis of correlation depicted in **Figure 5G** demonstrated a significant positive correlation between the main DE-DRGs and regulatory T cells, CD8 T cells, M0 macrophages, M1 macrophages, and resting dendritic cells. On the other hand, there was a significant negative correlation between the DE-DRGs and M2 macrophages, as well as resting mast cells. Subsequently, GSEA was applied to analyze the biological behavior of the two clusters. The GSEA enrichment analysis showed that the electron transport chain oxphos system in mitochondria, oxidative stress induced senescence, metabolic disorders of biological oxidation enzymes, triglyceride metabolism, diseases of programmed cell death and proinflammatory and profibrotic mediators were upregulated in cluster 1 (**Figure 5H**), all of which have been shown to be associated with the pathogenesis of NAFLD (39, 40). In conclusion, these results strongly indicated that the key DE-DRGs might be involved in the development and progression of NAFLD and were related to immune modulation.

### 3.5 Processing of the GSE136103 single-cell data set and construction of a cell atlas for NAFLD

**Figure 6:**
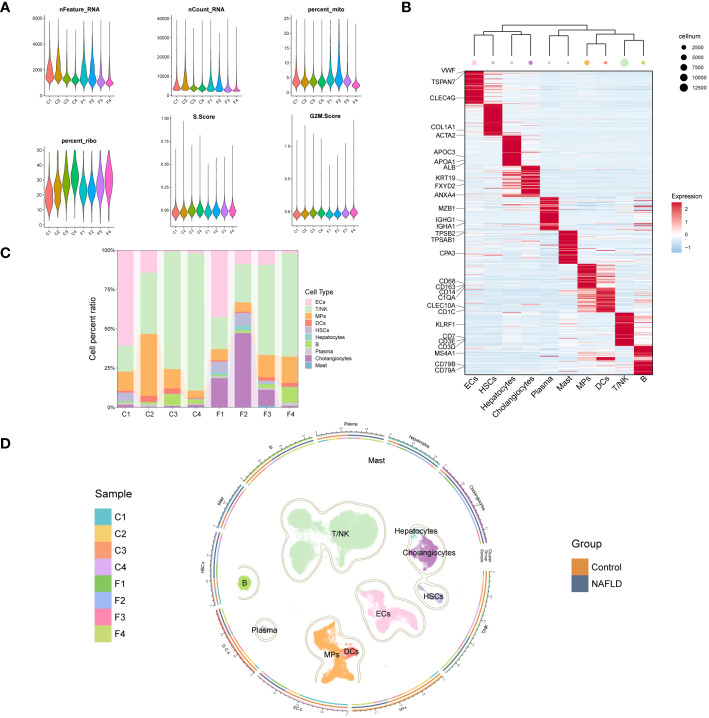
Clustering of GSE136103 scRNA-seq data and identification of cell types. **(A)** Violin plot of quality control parameters for scRNA-seq of liver tissue samples. **(B)** Heatmap of each cell cluster’s top 60 specific marker genes, while the upper panel of the heatmap shows the hierarchical clustering of clusters and the number of cells. In the heatmap, the gene expression is presented after Z-score processing, and the redder color represents the higher gene expression. **(C)** A bar chart shows the cell composition of cell clusters in 8 liver tissue samples. **(D)** UMAP plot (center) shows 23,929 cells from 8 samples, colored by major classes. The circle is divided into three parts from inside to outside, representing the distribution of samples in each cell cluster, the distribution of each cell cluster between groups, and the name of the cell clusters.

We further explored the immunomodulatory role of the key DE-DRGs in NAFLD patients through scRNA-seq data. After rigorous quality control screening, a total of 20896 genes and 23929 high-quality cells were included in the subsequent analysis (**Figure 6A**). The FindAllMarkers function and the Wilcoxon rank sum test were used to identify specific gene signatures for each cluster, as shown in **Figure 6B**. We finally identified 10 major cell lineages based on marker genes from previous studies and SingleR annotation, including T/NK cells (13083 cells, 54.7%, marked with CD3D, CD3E, CD7, and KLRF1), B cells (930 cells, 3.9%, marked with CD79A, CD79B, and MS4A1), plasma cells (286 cells, 1.2%, marked with IGHA1, IGHG1, and MZB1), MPs (3496 cells, 14.6%, marked with CD68, CD163, CD14, and C1QA), dendritic cells (DCs) (479 cells, 2.0%, marked with CD1C and CLEC10A), mast cells (57 cells, 0.2%, marked with TPSAB1, TPSB2, and CPA3), endothelial cells (ECs) (3475 cells, 14.5%, marked with CLEC4G, TSPAN7, and VWF), hepatic stellate cells (HSCs) (483 cells, 2.0%, marked with ACTA2 and COL1A1), hepatocytes (122 cells, 0.5%, marked with ALB, APOA1, and APOC3), and cholangiocytes (1518 cells, 6.3%, marked with ANXA4, FXYD2, and KRT19) (26–28). These ten populations contained cells from both controls and NAFLD patients’ liver tissues (**Figure 6C**). The cells were grouped into clusters using an unsupervised graph-based clustering method, which was visualized in the uniform manifold approximation and projection (UMAP) plot (**Figure 6D**).

### 3.6 The key DE-DRGs displayed significant differential expression in the MPs cluster

**Figure 7:**
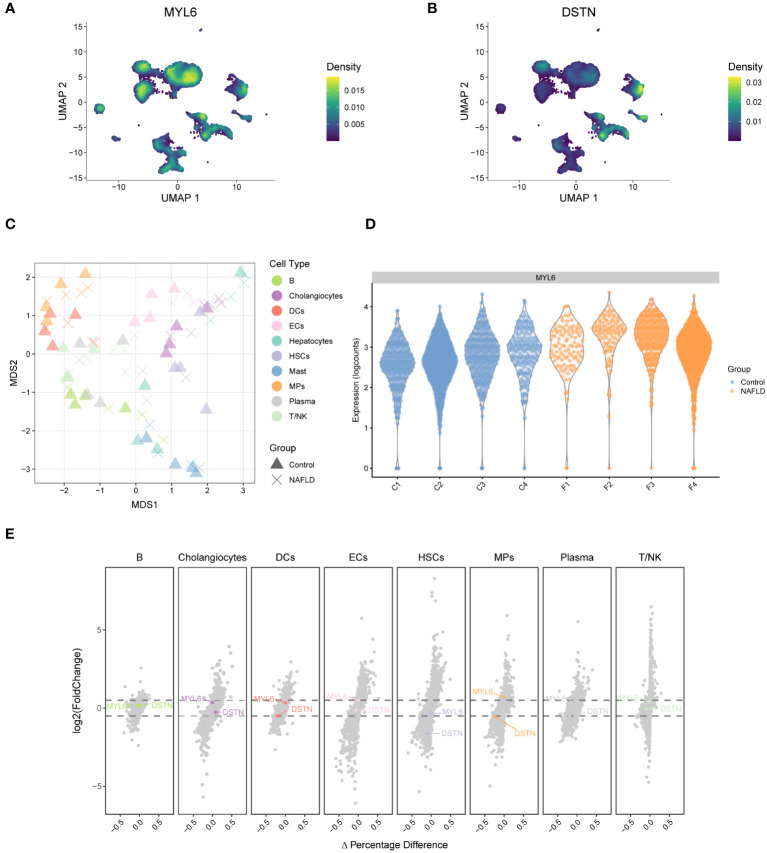
Weighted kernel density estimation of the key DE-DRGs and differential state analysis in GSE136103. **(A)** UMAP plot showing the Nebulosa expression densities for MYL6. **(B)** UMAP plot showing the Nebulosa expression densities for DSTN. **(C)** Pseudobulk-level multi-dimensional scaling (MDS) plot. **(D)** The violin plot shows the expression of MYL6 for the mononuclear phagocyte cluster. **(E)** The volcano plot shows the results of the differential state analysis in GSE136103 with the key DE-DRGs labeled.

A weighted kernel density estimation of the key DE-DRGs’ gene expression was performed using the Nebulosa (v1.10.0) package with default parameters. **Figure 7A** showed that MYL6 was expressed in most intrahepatic cells. In addition, DSTN was highly expressed mainly in cholangiocytes and ECs (**Figure 7B**). We generated a multi-dimensional scaling (MDS) plot using aggregated signal to explore similarities among samples. It is shown that the first dimension (MDS1) separates control and NAFLD samples, while the second dimension (MDS2) separates cell clusters (**Figure 7C**). To further clarify which cell clusters have altered the expression of key DE-DRGs in NAFLD patients compared to controls, we performed differential state analysis (**Figures 7D, E**; **Supplementary Table S8**). Interestingly, we found that MYL6 was significantly differentially expressed only in the MPs cluster (log2FC=0.597, *p* value=0.0124), and its expression trend was the same as in the bulk RNA samples (log2FC=1.247, *p* value<0.0001). In addition, DSTN was differentially expressed in the HSCs cluster (log2FC= -1.61, *p* value=0.00257) and MPs (log2FC= -0.543, *p* value=0.0298), but both showed the opposite trend of expression to that in bulk RNA samples (log2FC=0.596, *p* value<0.0001). Consequently, we focused on MYL6 for our subsequent investigation.

### 3.7 MYL6 might be involved in Kupffer cell polarization in NAFLD patients

**Figure 8:**
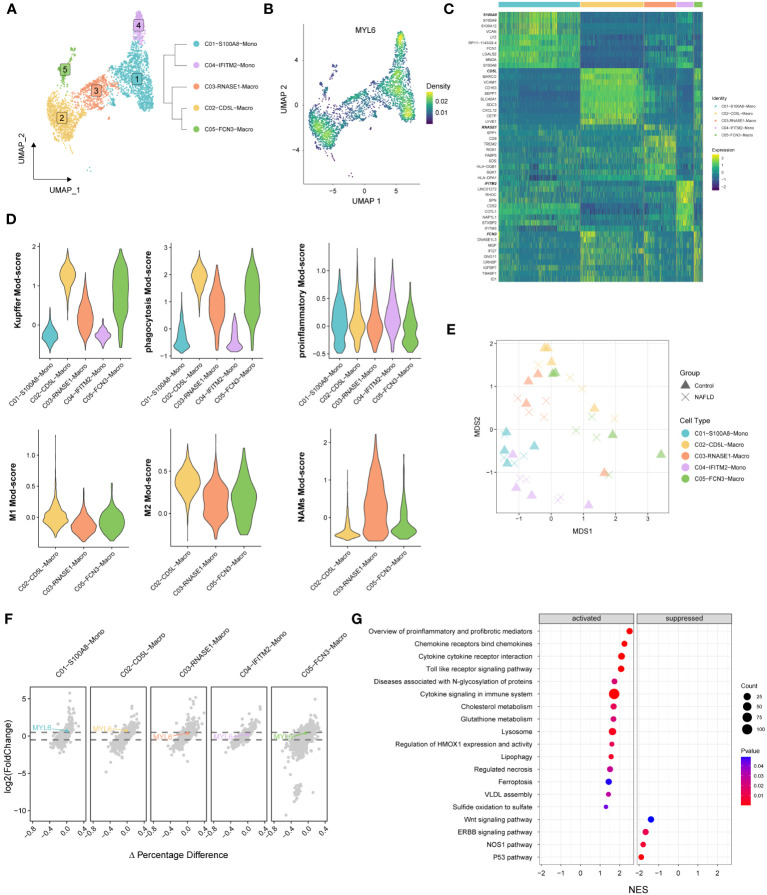
The specific phenotypes of mononuclear phagocytes. **(A)** UMAP plot showing 5 distinct clusters in mononuclear phagocytes, colored by cluster ID (left panel). A distance matrix generated in either gene expression space is used to infer the phylogenetic tree of 5 clusters. (right panel). **(B)** UMAP plot showing the Nebulosa expression densities for MYL6 in mononuclear phagocytes. **(C)** Heatmap of marker genes among different clusters. **(D)** Violin plots showing the functional module (Mod) scores of mononuclear phagocyte subsets. **(E)** Pseudobulk-level MDS plot. **(F)** The volcano plot shows the results of the differential state analysis in each mononuclear phagocyte cluster with MYL6 labeled. **(G)** GSEA in C02-CD5L-Macro cluster based on the canonical pathways gene sets.

We performed re-decimation clustering of MPs into 5 distinct clusters (**Figure 8A**; **Supplementary Figure S2**). It emerged 2 clusters for monocytes (with a suffix of Mono) and 3 clusters for macrophages (with a suffix of Macro). The phylogenetic tree relating to the “average” cell revealed that the 2 Mono clusters were closely related as a branch node, as were the 3 Macro clusters (**Figure 8A** right panel). MYL6 was highly expressed mainly in C01, C02 and C04 clusters (**Figure 8B**). Heatmap showed that C02-CD5L-Macro and C05-FCN3-Macro expressed high levels of Kupffer markers such as CD5L, MARCO, VCAM1, and CD163 (**Figure 8C**; **Supplementary Table S9**). In addition, by examining signature genes defined previously (28, 31, 32), we observed distinct functional status for each MPs subset, with the high Kupffer mod-score for C02-CD5L-Macro and C05-FCN3-Macro, high phagocytosis mod-score in the Macro clusters, similar proinflammatory mod-score in all clusters, highest M1 mod-score for C02-CD5L-Macro, and highest NASH-associated macrophages (NAMs) mod-score for C03-RNASE1-Macro (**Figure 8D**). Subsequently, differential state analysis in each cluster showed that MYL6 was significantly differentially expressed mainly in C01-S100A8-Mono (log2FC=0.524, *p* value=0.00489) and C02-CD5L-Macro (log2FC= 0.544, *p* value=0.02170) (**Figures 8E, F**; **Supplementary Table S10**). The results of GSEA enrichment analysis for C02-CD5L-Macro indicated that, in comparison to the control group, NAFLD patients exhibited up-regulation in the pro-inflammatory signaling pathway, the signaling pathways associated with regulated cell death (e.g., regulated necrosis and ferroptosis), and the signaling pathway related to sulfide metabolism (e.g., sulfide oxidation leading to sulfate) (**Figure 8G**; **Supplementary Table S11**). These results suggested that MYL6 might play an important role through M1 phenotype Kupffer cells in NAFLD patients.

### 3.8 The expression of MYL6 in patients with NAFLD and its close correlation with clinical parameters

**Figure 9:**
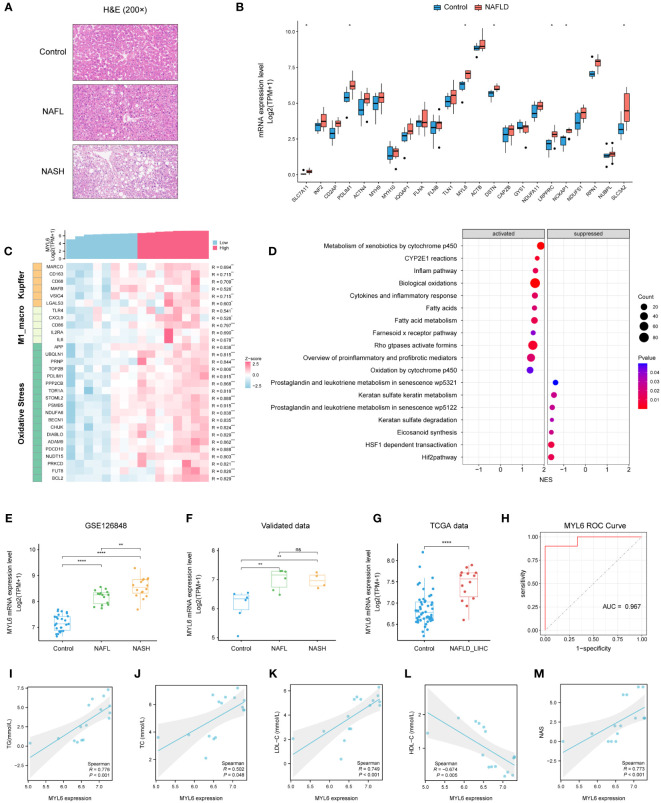
Validation of MYL6 in our data. **(A)** Liver tissue sections stained with H&E. **(B)** Relative expression of DRGs in liver samples from patients with NAFLD and controls. **(C)** Heatmap showing the MYL6 was significantly positively correlated with the expression of Kupffer cell signature genes, M1 phenotype macrophage signature genes, and oxidative stress-related genes. **(D)** GSEA in NAFLD patients based on the median MYL6 expression. **(E, F)** Box plot showing the expression of MYL6 among the control, NAFL, and NASH groups based on the different data. **(G)** Relative expression of MYL6 in liver tissue samples from patients with NAFLD-LIHC and controls. **(H)** Validation of MYL6 in NAFLD diagnosis in our data with ROC curve analysis. **(I–M)** Correlations of intrahepatic MYL6 mRNA levels with TG, TC, LDL-C, HDL-C, and NAS in our data. *P < 0.05, **P < 0.01, ***P < 0.001, ****P < 0.0001. ns: no statistically significant (P > 0.05).

We collected liver tissues from 16 patients and conducted HE staining as well as RNA-seq analysis (**Figure 9A**; **Supplementary Table S12**). The results indicated a substantial up-regulation of the majority of DRGs in the NAFLD group when compared to the control group (**Figure 9B**). In addition, the heatmap showed that MYL6 was significantly positively correlated with the expression of Kupffer cell signature genes, M1 phenotype macrophage signature genes, and oxidative stress-related genes (**Figure 9C**). Subsequently, we divided 10 NAFLD patients into high and low-expression groups based on the median MYL6 expression and performed GSEA between the two groups. The group with high expression of MYL6 showed an upregulation of inflammation-related pathways such as the inflame pathway, cytokines, and inflammatory response. They also showed an increase in fatty acid metabolism pathways and oxidative stress-related pathways (**Figure 9D**; **Supplementary Table S13**). It is noteworthy that there was a positive correlation between MYL6 expression and disease severity in NAFLD (**Figures 9E, F**). In addition, we downloaded data related to liver hepatocellular carcinoma (LIHC) from the TCGA database and categorized patients in whom the history of hepatocellular carcinoma risk factors was NAFLD only into the NAFLD-LIHC group. Our analysis showed that MYL6 expression was significantly higher in patients with NAFLD-LIHC than in controls (**Figure 9G**). Moreover, to validate the effectiveness of MYL6 in the diagnosis of NAFLD, ROC logistic regression analysis was performed based on our data. It was found that the AUC value of MYL6 was 0.967 (**Figure 9H**), indicating good sensitivity and specificity of MYL6 in the diagnosis of NAFLD. Further Spearman correlation analysis revealed that there was a significant positive correlation between intrahepatic MYL6 mRNA levels and NAS, as well as serum concentrations of TC, TG, and LDL-C. On the other hand, researchers discovered a negative connection between MYL6 expression and serum concentrations of HDL-C (**Figures 9I–M**).

## 4 Discussion

Due to the escalating prevalence of obesity and weight-related metabolic complications, NAFLD has become the most prevalent chronic liver ailment worldwide (41). In a subset of patients, hepatic steatosis progresses to NASH and may further develop into fibrosis, cirrhosis, and NAFLD-related HCC (42–44). Fatty acid metabolic reprogramming unique to hepatocytes is a key indicator of the liver cancer (45). Despite extensive research, the pathogenesis of NAFLD remains complex and not entirely understood, leading to a lack of effective therapeutic drugs, reliable non-invasive diagnostic tools, and dynamic biomarkers (46). Consequently, the identification of pathogenic targets and effective biomarkers for NAFLD is crucial for formulating personalized treatment strategies. There is no consensus on the role of disulfideptosis in the occurrence of NAFLD and its progression as a novel form of cell death (7). We aimed to investigate the potential role of DRGs in the pathogenesis of NAFLD and immune microenvironment based on single-cell and bulk RNA-seq.

In our study, a yellow module with 564 genes was identified, which has the highest correlation with the phenotype (NAFLD) in WGCNA. Furthermore, we identified 312 NAFLD-specific DEGs in DEGs and WGCNA, of which 293 were up-regulated and 19 were down-regulated. Functional enrichment analysis in the present study found that the NAFLD-specific DEGs were associated with important pathogenesis processes and pathways of NAFLD (39, 40), including Non−alcoholic fatty liver disease pathway, Oxidative phosphorylation pathway, and PPAR signaling pathways. These findings strongly indicated that the NAFLD-specific DEGs identified in this study play an important role in the occurrence and development of NAFLD and should be further investigated. Subsequently, immune infiltration analysis revealed significant alterations in the composition of immune cells in patients with NAFLD. The percentage of plasma cells, CD8 T cells, gamma delta T cells, M1 macrophages, and resting dendritic cells was higher in NAFLD samples compared to control samples, while a lower percentage of M2 macrophages was present. These were consistent with the results of previous studies (47–51). Although NAFLD is primarily a metabolic disorder, it also involves immune cell-mediated inflammatory processes. Inflammation becomes an indispensable factor in disease progression, particularly in the NASH stage. Liver immune cells are diverse in their steady state and undergo further evolution during NAFLD, directly impacting the severity of the disease (48).

To explore the role of disulfidptosis features in NAFLD, we examined the expression patterns of DRGs in the control and NAFLD groups. The results revealed that most of the DRGs were significantly differentially expressed in patients with NAFLD, which suggested a crucial role for DRGs in the occurrence and progression of NAFLD. On the other hand, we employed unsupervised clustering to categorize patients into two distinct disulfidptosis subtypes. We found that the key NAFLD-specific DE-DRGs (DSTN and MYL6) were up-regulated in cluster 1. Interestingly, cluster 1 has a high abundance of immune cell infiltration. GSEA analysis indicated that in cluster 1, oxidative stress-related pathways and pro-inflammatory pathways were upregulated. The expression levels of key DE-DRGs were positively correlated with the abundance of M1 macrophages and CD8 T cell infiltration. Prior studies have also found that disulfidptosis-related long non-coding RNAs are closely associated with the level of immune cell infiltration in liver tissue (52). Additionally, previous studies showed that elevated levels of reactive oxygen species played a crucial role in the inflammatory response, fibrosis, necrosis, and apoptosis occurring in NAFLD (53). Oxidative stress can induce Kupffer cells to produce various cytokines, such as TNF-α, thereby exacerbating inflammation and cell apoptosis (10). As for HSCs, their proliferation and collagen synthesis are triggered by lipid peroxidation induced by oxidative stress (54). This suggested that DRGs may promote immune cell infiltration in NAFLD through oxidative stress-related pathways, thereby accelerating the progression of the disease.

Subsequently, we further explored the expression distribution and biological functions of key DE-DRGs at the single-cell level within the liver. Traditional transcriptomic data from bulk RNA-seq pose challenges in depicting the heterogeneity of various cell types within the livers of both NAFLD patients and healthy individuals. With technological advancements, high-throughput sequencing techniques, such as scRNA-seq, have surfaced to furnish transcriptomic information at the cellular level (55, 56). We annotated and identified ten different cell subtypes based on scRNA-seq data. Results revealed that MYL6 was expressed in the majority of immune cells within the liver, while DSTN was predominantly expressed in cholangiocytes and ECs. Furthermore, MYL6 exhibited significant differential expression in the MPs cluster, with its expression trend aligning with that observed in bulk RNA samples. Subsequently, further refinement of the MPs cell subtype revealed that MYL6 was predominantly expressed in the CD5L-Macro resident in the livers of NAFLD patients. Functional module scores and enrichment analysis unveiled that the CD5L-Macro was characterized by high phagocytic activity and secretion of pro-inflammatory and fibrotic mediators, belonging to the M1 phenotype kupfer cells. GSEA enrichment analysis of C02-CD5L-Macro indicated activation of pro-inflammatory and pro-fibrotic signaling pathways, pathways related to regulatory cell death (such as regulated necrosis and ferroptosis), lipid metabolism pathways, and pathways associated with sulfur metabolism (such as sulfate formation due to sulfide oxidation) in NAFLD patients compared to the controls. Numerous studies suggested that Kupffer cells, a self-sustaining macrophage population residing in the liver, played a role in the progression of NAFLD (57, 58). During the onset of NAFLD, Kupffer cells underwent pro-inflammatory polarization, serving as crucial inflammatory mediators (51, 59). Subsequently, cytokines released by Kupffer cells stimulate a pro-inflammatory cascade, activating other immune cells (such as T cells), inducing the expression of transcription factors involved in lipid metabolism and transport, and promoting programmed cell death (60, 61). Therefore, we have reason to believe that MYL6 may contribute to the development of NAFLD through the promotion of M1 phenotype Kupffer cells.

Finally, we designed a validation study to investigate whether DRGs exhibited differential expression in NAFLD across 16 human liver tissue samples. Based on the RNA-seq results, the expression of the majority of DRGs was significantly increased in the NAFLD patients compared to the controls. In this study, particular emphasis was placed on the investigation of MYL6. Limited knowledge exists regarding the role of MYL6 in NAFLD. MYL6 encodes a myosin alkali light chain that is expressed in smooth muscle and non-muscle tissues, which is important for the assembly of cytoskeletal structures (62). Previous studies have identified a close association between MYL6 and the inflammatory disease Multiple Sclerosis, suggesting its predictive value for prognosis (63). Furthermore, MYL6 exhibited elevated expression in conditions such as obesity, asthma, and cervical cancer, yet the underlying mechanisms remain not fully elucidated (64). Our transcriptomic analysis revealed upregulation of MYL6 expression in the NAFLD group, closely associated with the expression of characteristic genes of Kupffer cells, M1 phenotype macrophages, and oxidative stress-related genes. The high-expression group of MYL6 exhibited upregulation of pathways related to inflammation, fatty acid metabolism, and oxidative stress. Intriguingly, the expression of MYL6 was significantly positively correlated with the progression of the disease spectrum in NAFLD. Evaluation of clinical features indicated a significant positive correlation between MYL6 expression levels and NAS, as well as concentrations of TC, TG, and LDL-C in the serum, while a significant negative correlation was observed with serum HDL-C concentration. Our findings suggested that MYL6 may promote the occurrence of oxidative stress, increase the infiltration of M1 phenotype Kupffer cells, and facilitate the progression of NAFLD through multiple pathways.

Certainly, this study inevitably has some limitations. The investigation solely validated the mRNA levels of DRGs and did not explore their protein-level changes in NAFLD. Additionally, further experimental validation was required to elucidate the potential regulatory role of DRGs in the occurrence and development of NAFLD. Finally, larger sample sizes in subsequent studies were needed to clarify the correlation between MYL6 expression levels and clinical pathological indicators of NAFLD.

In summary, this study comprehensively demonstrated the relationship between NAFLD and disulfidptosis using both single-cell and bulk RNA-seq data. It elucidated the regulatory role of DRGs in the hepatic immune microenvironment of NAFLD patients. Numerous potential target genes and pathways have been identified in NAFLD, providing potential targets for therapeutic interventions. Importantly, we revealed MYL6 as a novel immunomodulator participating in the pathogenesis of NAFLD. In conclusion, our research findings may offer a better understanding of the underlying mechanisms, thereby providing novel targets for the treatment of NAFLD.

## Data availability statement

The datasets presented in this study can be found in online repositories. The names of the repository/repositories and accession number(s) can be found below: GSE260666 (GEO).

## Ethics statement

The studies involving humans were approved by the Ethics Committee of the Second Xiangya Hospital of Central South University (No. 2019-050). The studies were conducted in accordance with the local legislation and institutional requirements. The participants provided their written informed consent to participate in this study.

## Author contributions

XL: Writing – original draft, Validation, Methodology, Investigation, Formal analysis, Data curation, Visualization. JG: Writing – original draft, Methodology, Investigation. HD: Writing – original draft, Resources, Data curation. ZH: Visualization, Software, Writing – original draft, Methodology. YW: Methodology, Investigation, Writing – original draft, Resources. ZS: Writing – review & editing, Supervision, Funding acquisition. JL: Writing – review & editing, Visualization, Supervision, Project administration, Funding acquisition, Conceptualization.
