# Supplementary material for: Unveiling the role of disulfidptosis-related genes in the pathogenesis of non-alcoholic fatty liver disease
Source: Front Immunol. 2024 May 15;15:1386905. doi: 10.3389/fimmu.2024.1386905 (PMC11133613; doi:10.3389/fimmu.2024.1386905)
Supplement: Supplementary file 1 [file DataSheet_1.docx]

Supplementary Material

Unveiling the role of disulfidptosis-related genes in the pathogenesis of non-alcoholic fatty liver disease

Xiaohua Luo^1^, Junjie Guo^1^, Hongbo Deng^1^, Zhiyong He^1^, Yifan Wen^1^, Zhongzhou Si^1*^and Jiequn Li^1*^

^1^Department of Liver Transplant, The Second Xiangya Hospital, Central South University, Changsha, China

*** Correspondence:** Zhongzhou Si: [zhongzsi@csu.edu.cn](mailto:zhongzsi@csu.edu.cn); Jiequn Li: [leejiequn@csu.edu.cn](mailto:leejiequn@csu.edu.cn)

# Supplementary Figures


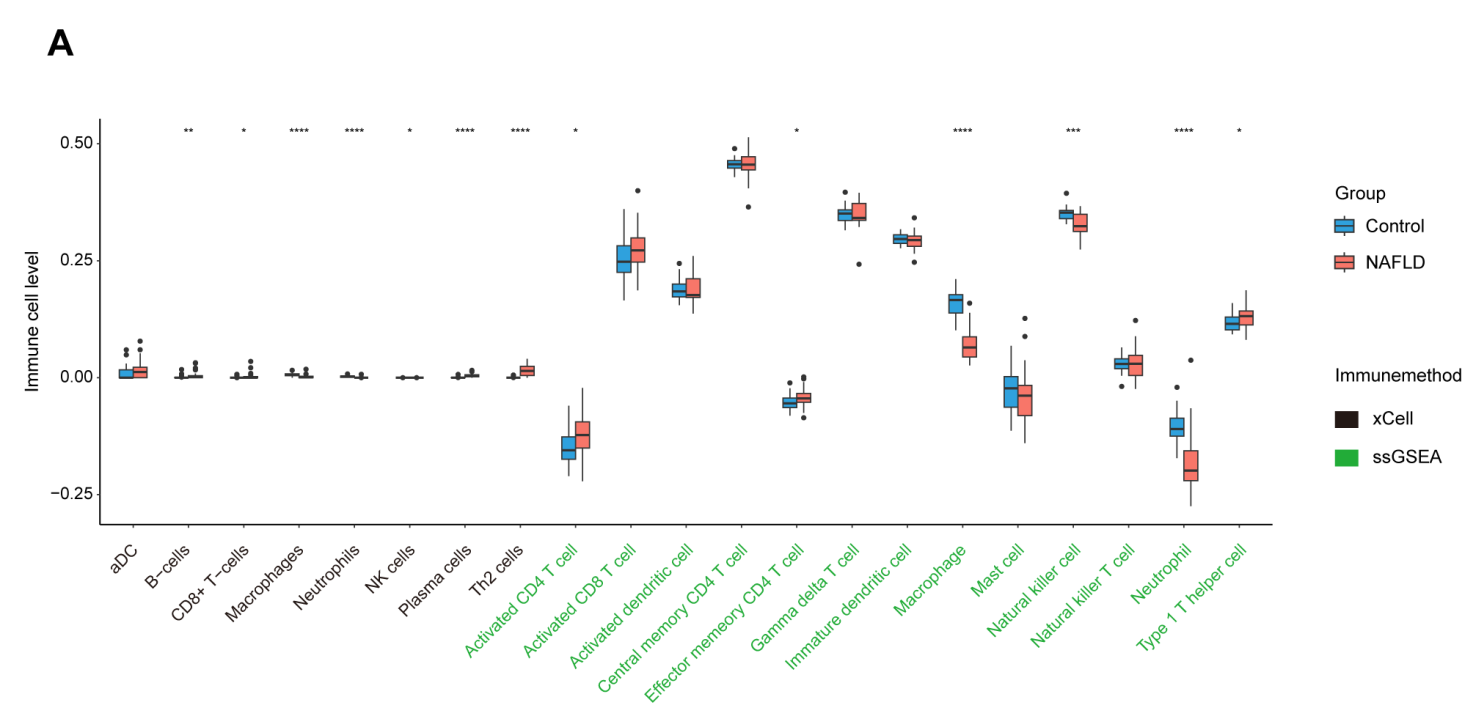


**Supplementary Figure 1. A box plot comparing the infiltration levels of immune cells between NAFLD patients and controls by xCell and ssGSEA algorithms.**

**
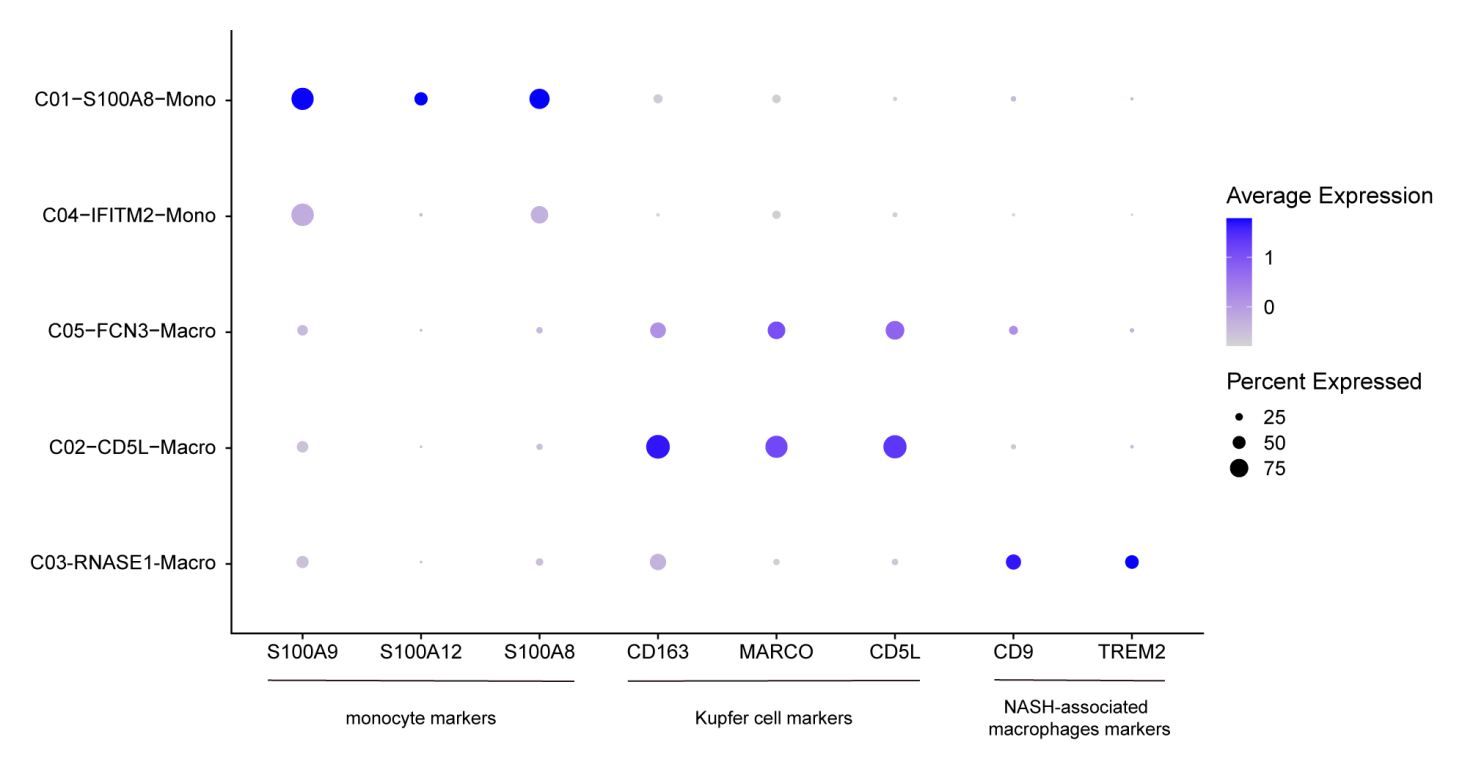
**

**Supplementary Figure 2. Dot plot of marker genes expression in MPs.**
